# Evaluating the use of lanthanide containing dendrimers for solvent paramagnetic relaxation enhancement

**DOI:** 10.1007/s10858-025-00468-9

**Published:** 2025-04-10

**Authors:** Westley Pawloski, James M. Gruschus, Ana Opina, Olga Vasalatiy, Nico Tjandra

**Affiliations:** 1 Biochemistry and Biophysics Center, National Heart, Lung, and Blood Institute, National Institutes of Health, 50 South Drive, Room 3503, Bethesda, MD 20892 USA; 2 Chemistry and Synthesis Center, National Heart, Lung, and Blood Institute, National Institutes of Health, Rockville, MD 20850 USA

## Abstract

Paramagnetic relaxation enhancement (PRE) is widely used in biomolecular NMR spectroscopy to obtain long-range distance and orientational information for intra- or intermolecular interactions. In contrast to conventional PRE measurements, which require tethering small molecules containing either a radical or paramagnetic ion to specific sites on the target protein, solvent PRE (sPRE) experiments utilize paramagnetic cosolutes to induce a delocalized PRE effect. Compounds developed as contrast agents in magnetic resonance imaging (MRI) applications typically consist of Gd chelated by a small molecule. Coordinating these Gd-containing small molecules to larger and inert scaffolds has been shown to increase the PRE-effect and produce more effective contrast agents in MRI. Inspired by their use as MRI contrast agent, in this work we evaluate the effectiveness of using a functionalized polyamidoamine (PAMAM) dendrimer for sPRE measurements. Using ubiquitin as a model system, we measured the sPRE effect from a generation 5 PAMAM dendrimer (G5-Gd) as a function of temperature and pH and compared to conventional relaxation agents. We also demonstrated the utility of G5-Gd in sPRE studies to monitor changes in the structures of two proteins as they bind their ligands. These studies highlight the attractive properties of these macromolecular relaxation agents in biomolecular sPRE.

**Supplementary Information:**

The online version contains supplementary material available at 10.1007/s10858-025-00468-9.

## Introduction

Paramagnetic ions found widespread use in NMR spectroscopy as a source for pseudocontact shift (PCS), relaxation enhancement (PRE), or residual dipolar coupling (RDC). Due to the relatively large gyromagnetic ratio of electrons relative to protons of ~ 655-fold, the effects of PRE and PCS can be observed to distances upwards of 30 Å and can be sensitive to low population states (Ravera et al. 2022; Koehler and Meiler 2011; Otting 2010). PRE and PCS effects in biomolecular NMR are typically achieved by covalently tethering a low molecular weight compound containing a paramagnetic probe to a reactive site on the biomolecule, such as a cysteine or reactive amine (Giannoulis et al. 2021; Roopnarine and Thomas 2024; Sjodt and Clubb 2017). Alternative attachment sites can be introduced through the use of synthetic amino acids or nucleotides (Faveri et al. 2024; Ledwitch et al. 2024). The two classes of paramagnetic agents often used are nitroxide radicals and paramagnetic metal ions which are chelated by 1,4,7,10-tetraazacyclododecane-1,4,7,10-tetraacetic acid (DOTA) or diethylenetriaminepentaacetic acid (DTPA) derivatives (Giovenzana et al. 2017; Anthis and Clore 2015). When chelated metals are used, the choice of ion determines whether the primary effect on the NMR resonance is paramagnetic relaxation enhancement (PRE) or pseudocontact shift (PCS), depending on the anisotropy of the magnetic susceptibility tensor of the metal center (Softley et al. 2020; Muntener et al. 2022; Vogel et al. 2021).

In a comparable manner, paramagnetic probes can be employed as cosolutes to provide a delocalized PRE effect. The magnitude of this solvent PRE (sPRE) is dependent on the average distance between the observable nuclei and the paramagnetic cosolute. Biochemical processes that expose or occlude the reporting nuclei would be detected as an increase or decrease in the solvent PRE, respectively (Lenard et al. 2022; Hocking et al. 2013). sPRE has been used to map the solvent accessible and electro-potential surface of proteins (Iwahara et al. 2023; Yu et al. 2024a, b; Kooshapur et al. 2018a, b; Linser et al. 2009; Otting* 2001; Mulder 2021; Yu et al. 2021a, b), aid in protein structure prediction, validation, and the detection of transient conformational states (Gong et al. 2017, 2018), and perform spectral editing by reducing the intensities of surface exposed resonances (Kellner et al. 2009).

sPRE is extensively applied in magnetic resonance imaging (MRI) to provide contrast between water in the vasculature and the surrounding tissues. Gd^3+^ chelated by DTPA was first approved for medical use as a contrast agent in 1988 and continues to be a primary component in MRI imaging. Its effectiveness arises from the large PRE effect of Gd³⁺, due to its seven unpaired electrons, which is rendered biocompatible by DTPA sequestering the ion (Iyad et al. 2023; Do et al. 2020; Kim et al. 2018; Rogosnitzky and Branch 2016). Efforts to produce more effective PRE agents have focused on tethering paramagnetic ions to high molecular weight species to increase relaxivity (the PRE effect per paramagnetic ion) and extend the residence time in the vasculature (Gallo et al. 2020; Lux and Sherry 2018; Wang et al. 2021). Polyamidoamine (PAMAM) dendrimer nanoparticles have been explored as drug delivery agents due to a highly controllable synthesis, tunable size, and easily modified spheroidal surfaces (Abedi-Gaballu et al. 2018; Chauhan 2018; Wang et al. 2022; Sarode and Mahajan 2024). Dendrimers of a desired generation are created by repeated nucleophilic substitution and deprotection reactions, starting from a core amine or polyamine (Bober et al. 2022; Fatemi et al. 2020; McMahon and Bulte 2018; Mekuria et al. 2016; Qiao and Shi 2015; Surekha et al. 2021; Longmire et al. 2008; Tomalia et al. 2007; Kobayashi and Brechbiel 2005). PAMAM dendrimers of 6 generations, functionalized with DOTA to chelate Gd³⁺, demonstrated an approximately 3-fold increase in relaxivity compared to both untethered DOTA and 2nd-generation dendrimers in vivo at low magnetic field (Wiener et al. 1994).

Here we are evaluating the effectiveness of using these PAMAM dendrimer contrast probes for biomolecular sPRE at higher fields. The schematic illustration for PAMAM dendrimer is shown in Fig. S1a. Each generation of dendrimer doubles the number of reactive terminal amines (G0 = 4, G1 = 8, G2 = 16, etc.). At the outset we expected that these dendrimers would provide an increase in relaxivity compared to small molecule cosolutes and that the DOTA-functionalized surface would avoid inducing any specific protein-dendrimer interactions. The sPRE performance of the fifth-generation PAMAM dendrimers (G5-Gd) on ubiquitin (Ub) were evaluated under variable dendrimer concentration, temperature, and pH. Additionally, secondary structure elements in urea-denatured Ub, which have previously been detected by sPRE (Kooshapur et al. 2018a, b), were probed with G5-Gd. Furthermore, the apo to holo transition of odorant-binding protein 44a (OBP44a) from fruit fly and the glutamine-binding protein (GlnBP) from *Escherichia coli* were detected with sPRE measurements using G5-Gd.

## Methods

### Preparation of lanthanide containing dendrimer

**Scheme 1:**
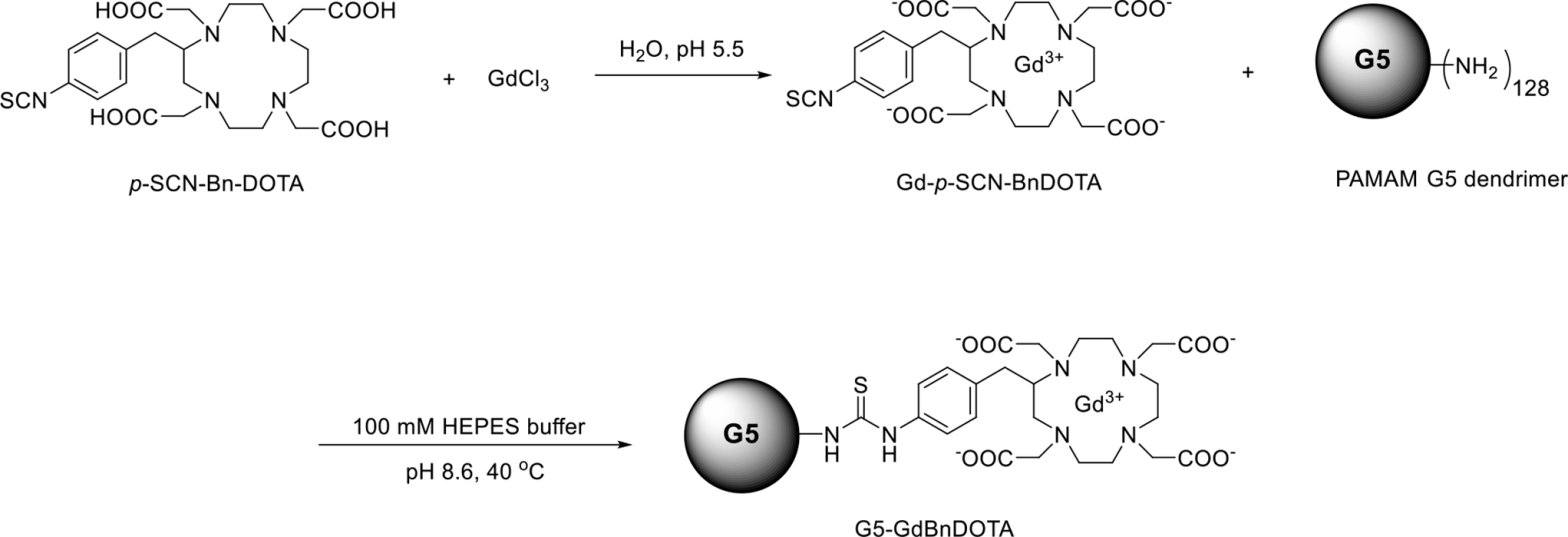
Synthesis of G5-GdBnDOTA conjugate

The G5-Gd (G5-GdBnDOTA conjugate) was prepared according to literature methods (Opina et al. 2015) as outlined in Scheme 1. Briefly, the gadolinium complex was prepared by mixing the equimolar amounts of the gadolinium chloride and the ligand (p-NCS-Bn-DOTA) in deionized water. The pH of the reaction was maintained at 5.5 with 1 M NaOH. Upon observation of no pH change, the pH of the reaction was adjusted to 7.4–7.6. The solution was filtered and lyophilized. The complex (128 eq) was added to the G5 dendrimer solution (10 mg/mL, 100 mM HEPES, pH 8.6). The reaction was stirred for 24 h at 40 C. The conjugate was purified using Amicon-Ultra centrifugal filter unit (30 kDa cutoff) with deionized water (5 times). The conjugate was collected from the unit and lyophilized. The number of complexed gadolinium per dendrimer was calculated based on the CHN elemental analysis and gadolinium Inductive Coupled Plasma Mass Spectrometry (ICP-MS) results. Briefly, elemental analysis found for the G5 dendrimer conjugate is C 38.1% and Gd 13.7% or 38.40 total carbon atoms per gadolinium atom. The gadolinium complex formula C_24_H_30_N_5_O_8_SGd is equivalent 14.40 carbon atoms of dendrimer per gadolinium complex. The dendrimer formula C_1264_H_2532_N_506_O_252_ gives a ratio of 1264/14.4 or approximately 88 Gd chelates per dendrimer.

### NMR samples

D_2_O was added to all NMR samples to 5% v/v (Hocking et al. 2013). ^15^N-labeled Ub was prepared as previously described (Strickland et al. 2017) and exchanged into 50 mM sodium phosphate pH 5.6. For other pH values 50 mM sodium phosphate buffers were prepared at the desired pH and samples were buffer exchanged using an Amicon filter with a 3 kDa cutoff. For experiments with denatured Ub samples were buffer exchanged into 8 M urea, 10 mM glycine pH 2.5. NMR samples of native and urea-denatured Ub were prepared at 400 µM.

^15^N-labeled OBP44a was prepared as described previously (He et al. 2023a, b). The apo form of OBP44a was purified using reverse phase HPLC as the final step to ensure no residual fatty acid or lipid was bound to the protein during the expression and purification. The holo-form of OBP44a was created by adding 8(Z)-eicosenoic acid (8(Z)-C20:1) fatty acid at a 1.1:1 ratio to the protein sample. 400 µM NMR samples were prepared in 20 mM postassium phosphate pH 6.6. Wild type (Hocking et al. 2013)^15^N-labeled GlnBP was expressed and purified following a protocol described in Bermejo et al. (Bermejo et al. 2009) Since the wild-type protein does not contain any cysteine, reducing agent was left out of the purification. To obtain ligand free protein the sample was extensively dialyzed against 6 M Urea and refolded by dialysis against 20 mM sodium phosphate pH 7.5. L-glutamine was added to 1.1 equivalents to generate the holo-form. NMR samples were prepared at 500 µM.

G5-Gd was prepared by hydrating the lyophilized powder to a final Gd concentration of 10 mM or 50 mM. Gadobutrol (Gadovist ^®^, Bayer, Berlin, Germany) was used to create sPRE to which the G5-Gd data can be compared to. Gadobutrol was prepared as 10 mM or 50 mM solutions by diluting the 1 M gadobutrol stock solution, which contains calcobutrol sodium and trometamol, with water and was added into the NMR sample without further modification of the stock solution.

### NMR experiments

The sPRE was calculated by taking the difference in the (Ravera et al. 2022)^1^H R_2_ relaxation rate of the protein sample with and without the G5-Gd or Gadobutrol. PRE rates were measured for backbone amide protons using a two-time-point (Hocking et al. 2013)^15^N-HSQC-based interleaved experiment (Iwahara et al. 2007). Errors in the PRE are propagated from the estimated noise in the diamagnetic and paramagnetic spectra. Measurements were performed on a Bruker Avance 600-MHz spectrometer equipped with a cryo-probe and Z-pulsed field gradient. Spectra were processed with NMRPipe (Delaglio et al. 1995) and analyzed with CCPNMR V3^54^. Ub PRE was measured with 0.57 µM G5-Gd (50 µM Gd) at 298 K unless otherwise specified, OBP44a PRE with 11 µM G5-Gd (1mM Gd) at 298 K, and GlnBP PRE with 23 µM G5-Gd (2mM Gd) at 310 K.

## Results

**Fig. 1:**
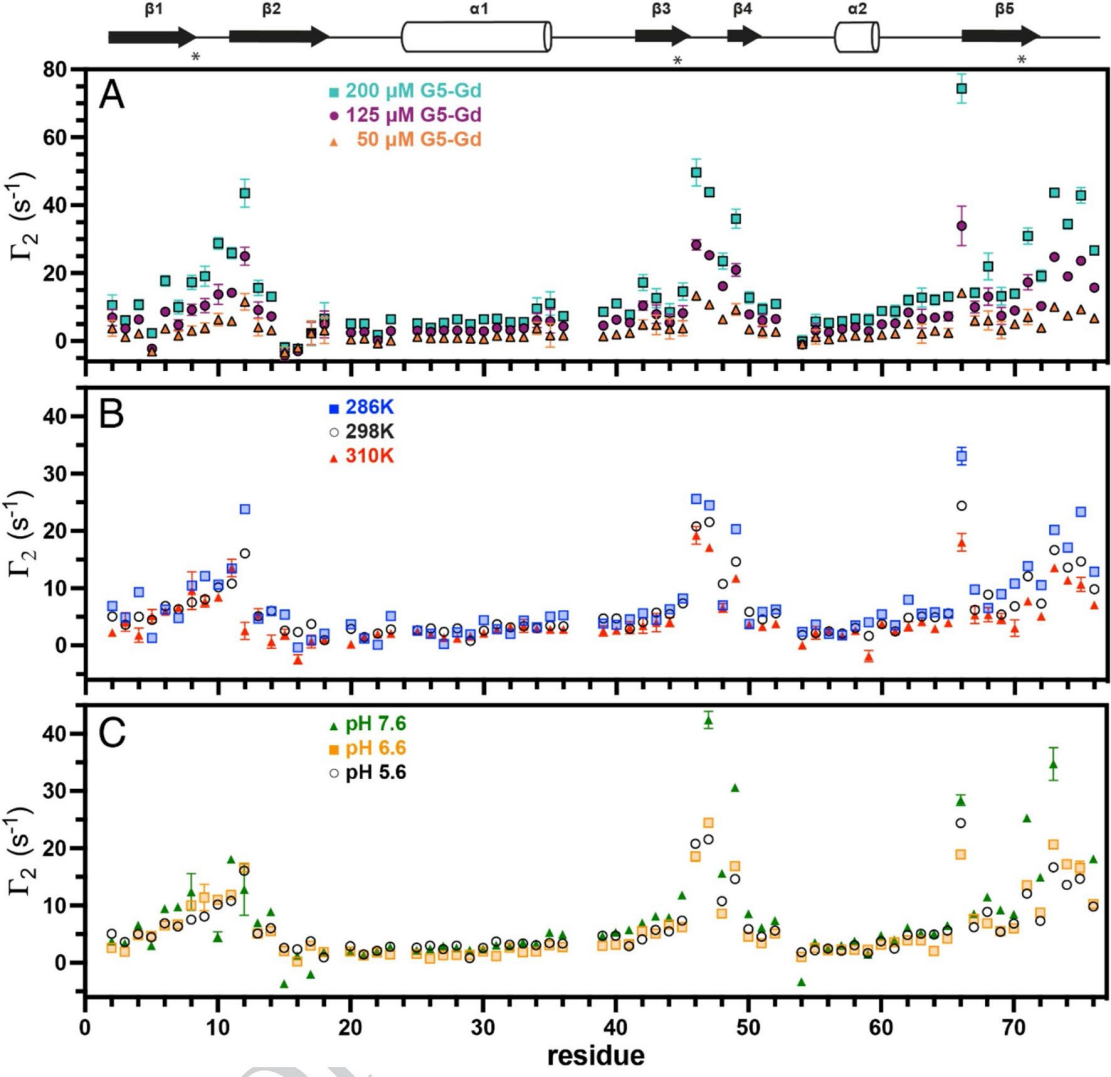
^1^H_N_ transverse sPRE rates of ubiquitin backbone amides by fifth-generation PAMAM dendrimer (G5-Gd). **A**) )^1^H_N_ Γ_2_ data for G5-Gd at Gd^3+^ concentrations of 50 µM (orange), 125 µM (purple), and 200 µM (cyan). Hydrophobic patch residues are indicated by an asterisk. **B**) )^1^H_N_ Γ_2_ data at 286 K (blue), 298 K (black), and 310 K (red). **C**) )^1^H_N_ Γ_2_ data at pH 5.6 (black), pH 6.6 (light orange), and pH 7.6 (green)

The per-residue PRE for Ub with G5-Gd is illustrated in Fig. 1a with three concentrations of the PRE agent. The amide relaxivity profile is comparable to previously reported sPRE data for Ub with DTPA-Gd³⁺ (Yu et al. 2024a, b) or proxyl nitroxides (Yu et al. 2021a, b). The largest PRE with G5-Gd occurs at T66, followed by residues proceeding the Ub hydrophobic patch residues of L8, I44, and V70. The residues with an elevated PRE are mapped on the structure of Ub in Supplementary Fig. S1b. The relaxivity, as measured by an increase in R_2_, was linear with G5-Gd concentration, as expected (Fig. S1c). To evaluate the impact of temperature on relaxivity, experiments were conducted at 286 K, 298 K, and 310 K, and Fig. 1b demonstrates the inverse relationship of the G5-Gd PRE and temperature for most residues. In addition, the effect of pH was tested by varying values from pH 5.6 to 6.6 and 7.6 and these data are plotted in Fig. 1c. Similar to the temperature dependence, substantial increases in the PRE at the higher pH are localized to residues which exhibit a large PRE at pH 5.6.

**Fig. 2:**
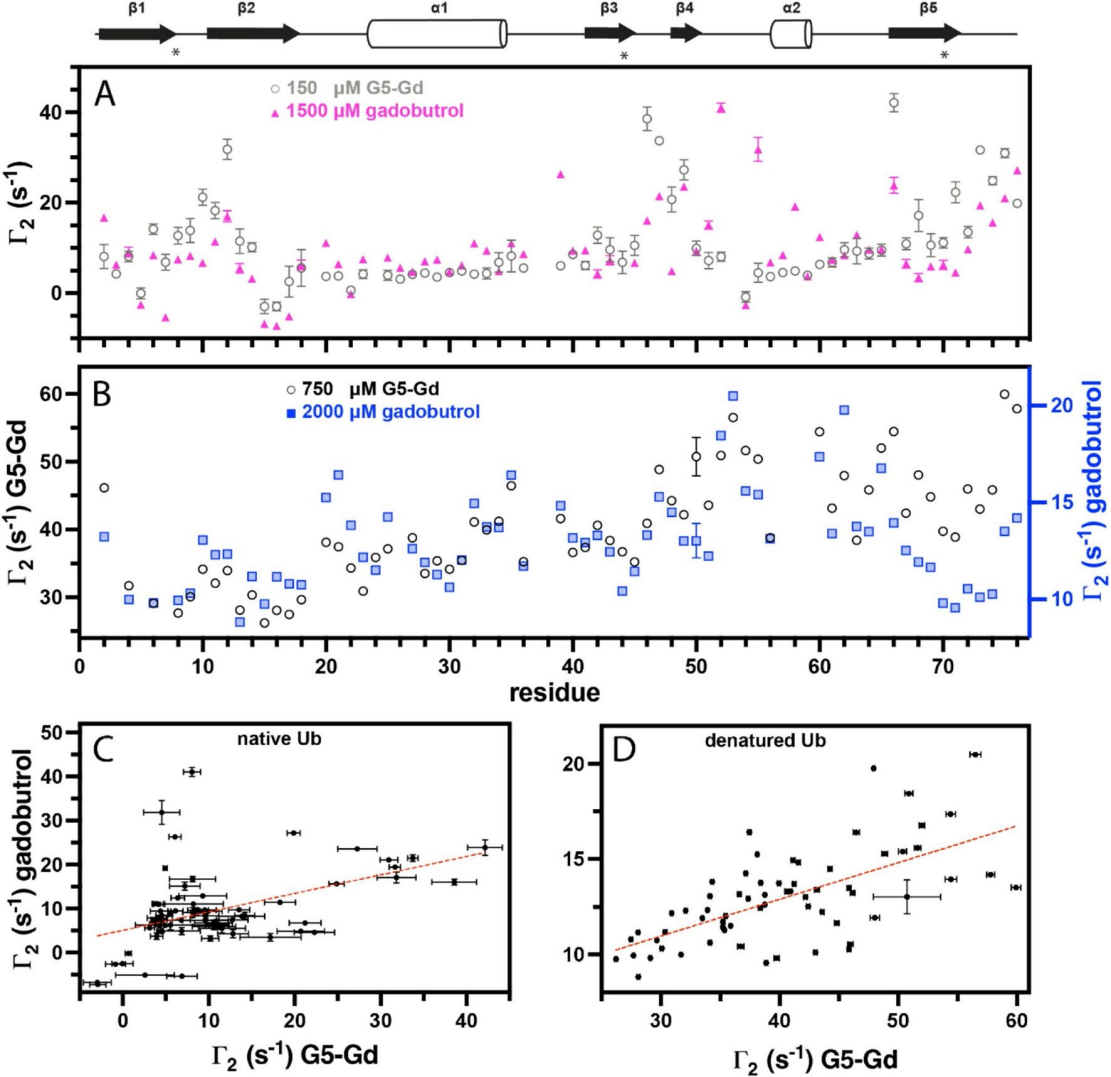
^1^H_N_ transverse sPRE rates of ubiquitin backbone amides under native and denaturing conditions with fifth-generation PAMAM dendrimer (G5-Gd) and gadobutrol. Hydrophobic patch residues are indicated by an asterisk. The concentration of Gd^3+^ is indicated for both compounds. **A**) )^1^H_N_ Γ_2_ data for native Ub with G5-Gd (grey) and gadobutrol (pink). **B**) )^1^H_N_ Γ_2_ data for urea-denatured Ub with G5-Gd (black) and gadobutrol (blue). Correlation analyses for the PRE from experiments with G5-Gd and gadobutrol for native Ub (**C**) and denatured Ub (**D**). The linear fits are represented with a dashed red line and the correlation coefficents are 0.48 for native Ub and 0.64 for denatured Ub

To assess the per-ion gain in R_2_ relaxivity of G5-Gd vs. conventional PRE agents, the sPRE of Ub was compared between 150 µM of Gd^3+^ in G5-Gd (corresponding to a dendrimer concentration of 1.70 µM) to 1500 µM of Gd^3+^ in gadobutrol, a monomeric DOTA-like chelator, a 10-fold increase in concentration. The overlay of this data presented in Fig. 2a shows that G5-Gd induces a roughly 10-fold larger per-ion PRE overall, though for residues more protected from PRE, in α-helices for example, the effect is less pronounced. Interestingly, under our test conditions gadobutrol induces a pronounced PRE in the two loops between the α-helix and β-strand 3 and between β-strand 4 and the 3_10_-helix (Fig. S1b). Elevated PRE in these two loops has also been observed when DTPA-Gd^3+^ is used as the sPRE cosolute (Kooshapur et al. 2018a, b).

Fine structural details, such as residual secondary structure in denatured proteins, can be gleaned from sPRE measurements and have been previously detected in urea-denatured Ub using a DTPA paramagnetic agent (Kooshapur et al. 2018a, b). To investigate the impact of the size and accessibility of the sPRE agent on detection of residual structural elements, the experiment was repeated using gadobutrol and G5-Gd and the results are shown in Fig. 2b. The PRE profile obtained from gadobutrol closely resembles the one observed with DTPA and shows a reduced PRE for residues near secondary structure elements within the folded protein. The PRE profile recorded with G5-Gd demonstrates a similar trend but shows larger values for the 10 residues at the C-terminus. This elevated PRE is not associated with chemical shift perturbations, which likely rules out a specific binding interaction. The relaxivity of G5-Gd was about six times greater than gadobutrol in these experiments. The correlation plots between sPRE obtained using gadubutrol and G5-Gd for native and denatured ubiquitin are shown in Fig. 2c & d, respectively. Some large PRE values for gadubutrol in native ubiquitin are not observed in G5-Gd (Fig. 2c), perhaps due to some non-specific interaction of the small gadubutrol to ubiquitin.

**Fig. 3:**
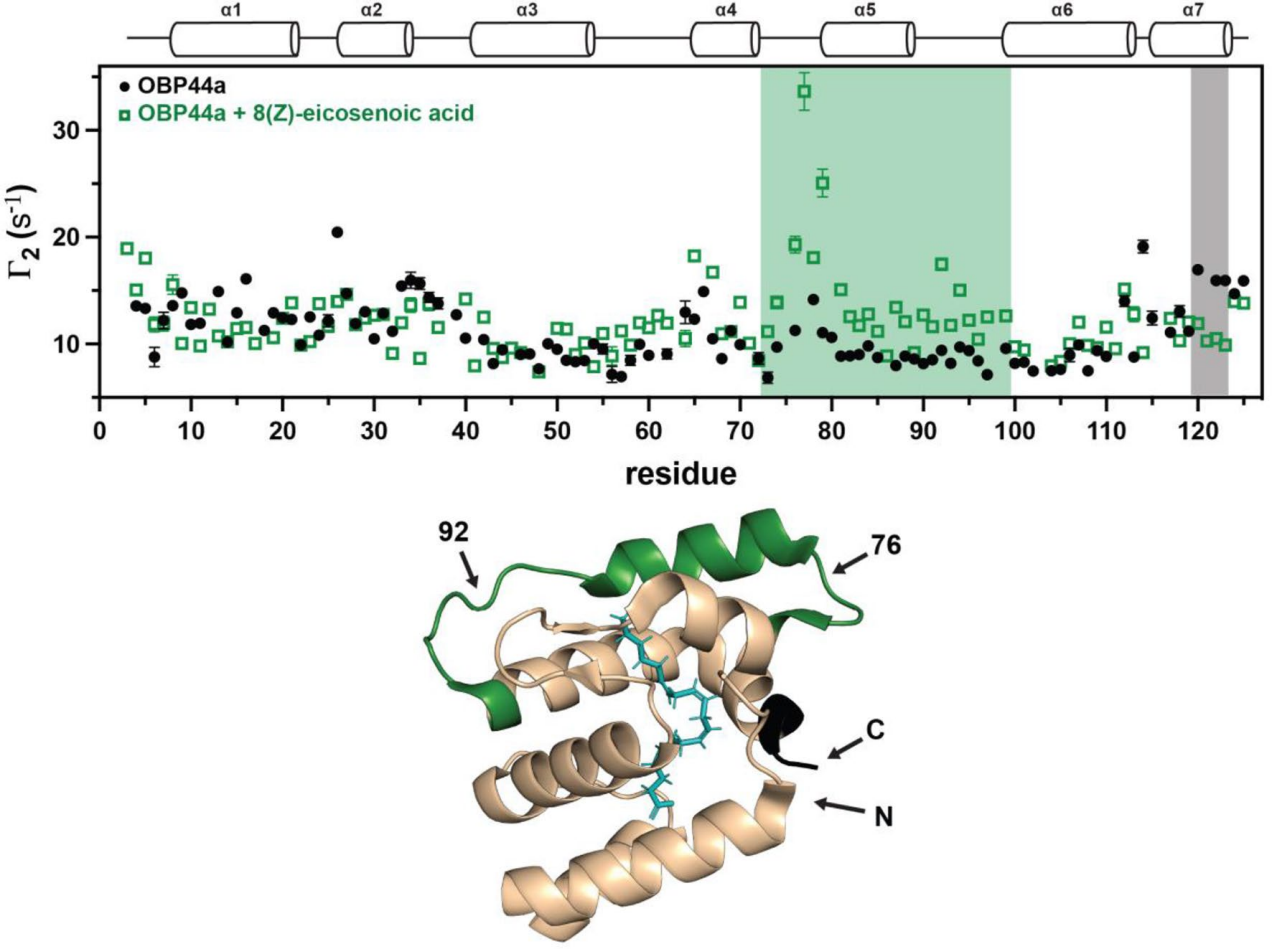
^1^H_N_ transverse sPRE rates Γ_2_ of odorant-binding protein (OBP44a) with 11 µM G5-Gd (1mM Gd) in the apo (black) and holo (green) form with 8(Z)-eicosenoic acid. Top panel: Overlay of the two datasets with regions that have consistently larger PRE for either the free or bound states shaded with the respective colors. Bottom panel: Structure of OBP22a bound to arachidonic acid (PDB: 6NBN) which is homologous to OBP44a bound to a fatty acid. Key residues are highlighted onto the structure: residues in black have larger PRE in the apo form while residues in green exhibit larger PRE in the holo form. Arachidonic acid is colored cyan

The effectiveness of G5-Gd as a sPRE agent for detecting global alterations in protein structure was evaluated through the investigation of ligand-induced conformational changes in two model systems. OBP44a is a lipid chaperone protein from *Drosophila melanogaster* that binds to long chain fatty acids (He et al. 2023a, b). A recent report indicated that the C-terminus undergoes a transition from a random coil to ⍺-helical secondary structure upon binding to these ligands, and this change probably assists with capping the binding pocket (Cotten et al. 2024; Wang et al. 2020). The transition of OBP44a from apo to holo with 8(Z)-C20:1 was evaluated by sPRE with G5-Gd, and the data are overlayed in Fig. 3. Note that the data were collected in the presence of only 11 µM of Gd-containing dendrimer (1mM Gd ions). When comparing the two datasets two regions show notable differences in PRE profiles. Residues 120–124 near the C-terminus exhibit a reduced PRE in the bound form, likely due to the C-terminus folding into the more compact α-helical structure. In contrast, the stretch of residues from 74 to 99 shows a larger PRE in the bound state compared to the apo form, with residues 74–81 having an especially pronounced increase in PRE. These latter residues were shown to be perturbed in crystal structures of OBP22, a homologous protein, when bound to various ligands (Wang et al. 2020), but the role of these residues in ligand binding has not been elucidated.

**Fig. 4:**
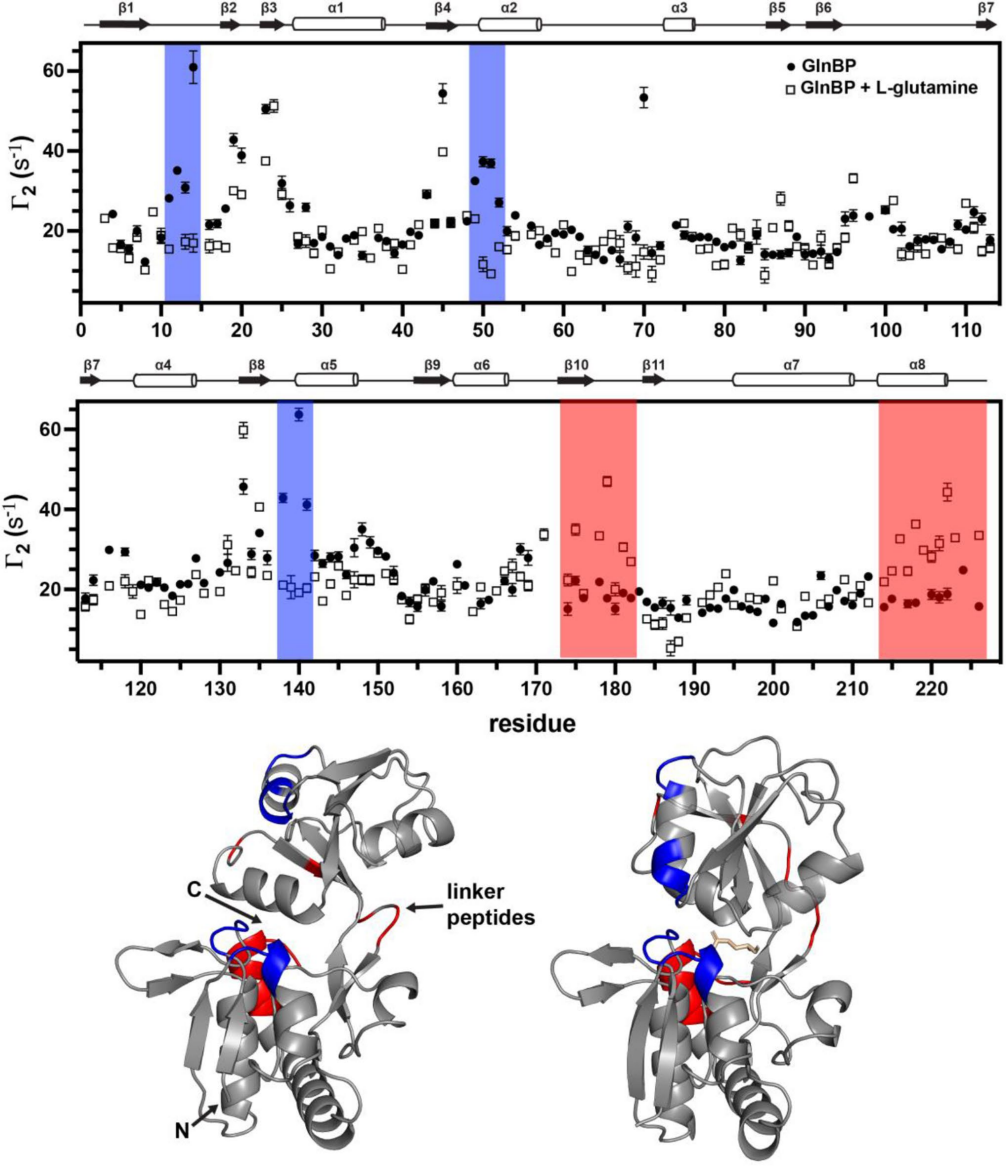
^1^H_N_ transverse sPRE rates Γ_2_ of glutamine binding protein (GlnBP) with 23 µM G5-Gd (2mM Gd) in the apo (filled circles) and holo (open squares) forms. Top two panels: Overlay of the two datasets with regions that have a substantially larger PRE for either the free or bound states shaded blue or red, respectively. Bottom panel: Structures of GlnBP in the apo (PDB: 1GGG) and holo (PDB: 1WDN) forms, positioned left and right, respectively. Key residues are highlighted on each structure: residues in blue have larger PRE in the apo form while residues in red exhibit larger PRE in the holo form. L-glutamine is colored beige

GlnBP is an essential component for nutrient uptake in Gram-negative bacteria that is highly specific for binding L-glutamine. As a member of the periplasmic binding protein (PBP) family GlnBP consists of two globular domains connected by two polypeptide linker segments that can adopt an “open” conformation, where the interdomain region is solvent and solute accessible, and a “closed” conformation where the two domains converge on the ligand (Bermejo et al. 2010; Davidson et al. 2008). The transition from the open to closed conformation upon binding L-glutamine was examined by sPRE with G5-Gd and the data are overlayed in Fig. 4. These data were obtained with 23µM of Gd-containing dendrimer (2mM Gd ions). Residues 11–14, 49–52, and 138–141 have elevated PRE values in the open state; these residues are located on the solvent-facing side of the binding pocket which becomes occluded upon L-glutamine binding. In contrast, residues within the range of 174–182 and the 13 C-terminal residues are situated on the opposite side of the linker peptides and are partially solvent accessible in the open configuration. After the globular domains rigidify around the L-glutamine ligand these residues become locked in a solvent-exposed conformation (Fig. 4).

## Discussion and conclusions

sPRE studies typically utilize high concentrations of either nitroxide radicals or lanthanide ions protected by a moiety that limits specific interactions with biomolecules. In the context of MRI, large complexes which are functionalized with these same molecules have been shown to induce a larger relaxivity relative to the monomeric species (Tang et al. 2013). PAMAM dendrimers have been extensively cited as ideal scaffolds for building such compounds due to the straightforward synthesis of high molecular weight derivatives and ease of modifying the surfaces (Venditto et al. 2005). The increase in the observed relaxivity was attributed to the increased molecular weight, thus correlation time of the contrast agent. Whether this increase in relaxivity will hold true at very high magnetic fields was not clear.

Although these compounds remain experimental in the clinic, based on our preliminary results PAMAM dendrimers could be attractive agents for sPRE in NMR spectroscopy. Using Ub as an example, we have demonstrated that these dendrimers functionalized with DOTA-Gd^3+^ groups enhance the transverse PRE effect upwards of 10-fold per Gd^3+^ ion compared to a small molecule probe. Unsurprisingly, the residues with large PRE values are located in highly dynamic regions adjacent to the Ub hydrophobic patch (Lange et al. 2008) except, anomalously, for T12 and T66. The PRE with G5-Gd was shown to be inversely correlated with temperature, likely due to Curie spin relaxation and favorable water exchange kinetics at lower temperature. This behavior has previously been demonstrated in transverse relaxation enhancement induced by PRE compounds with a coordinated water molecule (Caravan et al. 2001; Strickland et al. 2016), as is the case with DOTA chelates. Residues with significant PRE at pH 5.5 also exhibit increased relaxation at higher pH, with some of these residues showing fast solvent exchange rates at pH 5.8 (Brand et al. 2007; Jahr et al. 2011) and 7.0.(Jurt & Zerbe 2012). In contrast, the amides of T12 and T66 are not adjacent to residues with a fast solvent exchange rate yet exhibit a large sPRE. This suggests that the observed PRE enhancement is additionally influenced by water exchange kinetics at the dendrimer rather than solely backbone amide exchange (Tóth et al. 1996).

sPRE experiments typically utilize small and hydrophobic paramagnetic agents which, by nature of their compact geometry, can sample the majority of the solvent-exposed protein surface. By moving to the larger spheroid structure of G5-Gd some of these cavities could become occluded to the paramagnetic species and exhibit a reduced PRE. Despite the large size of G5-Gd, the PRE profile of denatured Ub overall resembled those obtained with DTPA or gadobutrol; however, an elevated PRE near the C-terminus suggested possible non-specific interactions with some of these residues. In experiments with native Ub a cluster of residues exhibited substantial PRE with gadobutrol and DTPA but not with G5-Gd (Fig. S1b.) and could reflect either the larger size of G5-Gd or weak interactions facilitated by the smaller size of these probes. sPRE experiments using the ligand-binding proteins OBP44a and GlnBP demonstrated that G5-Gd is capable of detecting subtle tertiary structural changes induced by ligand binding.

Together these data show that PAMAM dendrimers can be functionalized into potent sPRE agents for NMR spectroscopy which yield large increases in relaxivity relative to commonly used PRE probes. Similar to the observed increase of relaxivity in low field MRI, we also saw a notable increase at very high magnetic field. In this work the dendrimers were capped with Gd^3+^ chelated by benzyl-DOTA tag which is known to be a relatively inert chemical probe. While this compound induced similar relaxation profiles to gadobutrol, a small molecule analogue of the DOTA functional group, there were discrepancies that could be attributed to the difference in size of the particle, affecting non-specific interactions with the analyte, or the water dynamics at the chelating groups. At the lower concentrations required (1–25µM) to achieve a sufficient PRE for analysis any non-specific interactions with these dendrimers would be minimized, and dendrimer-based PRE could be particularly useful for detecting exceptionally weak interactions or for increasing the local concentration of other functional groups.

## Electronic supplementary material

Below is the link to the electronic supplementary material.

Supplementary Material 1

## Data Availability

No datasets were generated or analysed during the current study.
