# Supplementary material for: Evaluating the use of lanthanide containing dendrimers for solvent paramagnetic relaxation enhancement
Source: J Biomol NMR. 2025 Apr 10;79(3):199–208. doi: 10.1007/s10858-025-00468-9 (PMC12378495; doi:10.1007/s10858-025-00468-9)
Supplement: Supplementary file 1 — Supplementary Material 1 [file 10858_2025_468_MOESM1_ESM.docx]

**Evaluating the use of lanthanide containing dendrimers for solvent paramagnetic relaxation enhancement**

Westley Pawloski^1^, James M. Gruschus^1^, Ana Opina^2^, Olga Vasalatiy^2^, Nico Tjandra^1*^

^1^Biochemistry and Biophysics Center, National Heart, Lung, and Blood Institute, National Institutes of Health, Bethesda, MD, 20892, USA.

^2^Chemistry and Synthesis Center, National Heart, Lung, and Blood Institute, National Institutes of Health, Rockville, MD, 20850, USA.

**Supplementary Information**

***Corresponding Author:**

Nico Tjandra

Biochemistry and Biophysics Center

National Heart, Lung, and Blood Institute

50 South Drive, Room 3503

Bethesda, MD 20892, USA

Telephone: (301) 402-3029

Telefax: (301) 402-3405

e-mail: [tjandran@nhlbi.nih.gov](mailto:tjandran@nhlbi.nih.gov)


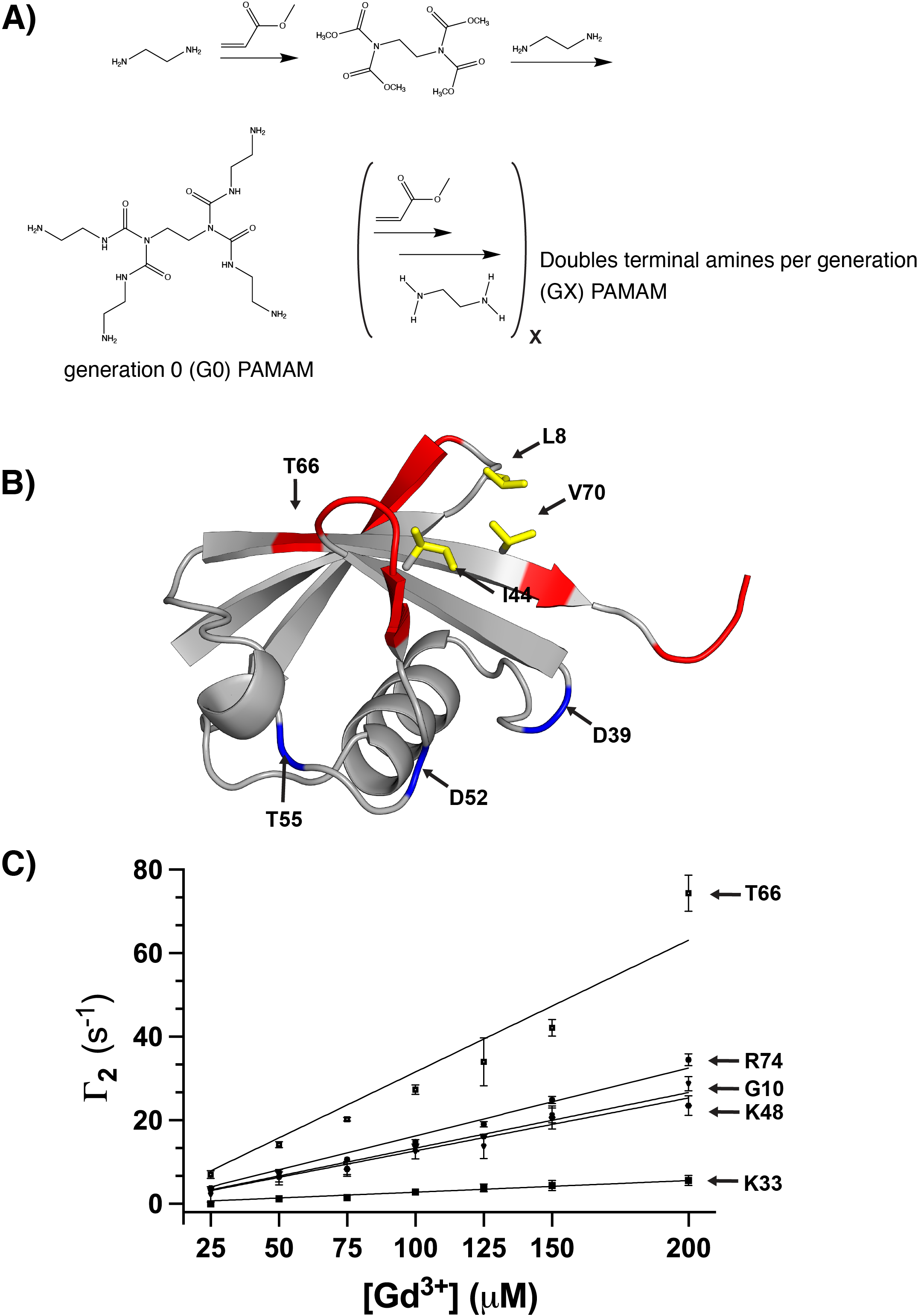


**Figure S1.** PAMAM dendrimer, ubiquitin PRE profile and linear regression for select residues. **A)** Schematic of PAMAM core and reaction steps to form the dendrimer. The number of terminal amines doubles for each generation. **B)** Structure of ubiquitin (Ub) (PDB: 1UBQ) with hydrophobic patch residues highlighted with yellow sidechains. Residues with the largest PRE are highlighted in red. Residues highlighted in blue have a large PRE with gadobutrol only. **C)** Linear regression fit of ^1^H_N_ transverse sPRE for select residues as a function of G5-Gd concentration.
